# Development of Toughened Recycled Polyethylene Terephthalate and Micronized Rubber Composites for 3D Printing Applications: Compatibilization Strategies and Performance Assessment

**DOI:** 10.1021/acsomega.4c10726

**Published:** 2025-05-01

**Authors:** Aboulfazl Barati, Deacon S. Godfrey, Erfan Dashtimoghadam

**Affiliations:** Center for Materials and Manufacturing Sciences, Departments of Chemistry and Physics, Troy University, Troy, Alabama 36082, United States

## Abstract

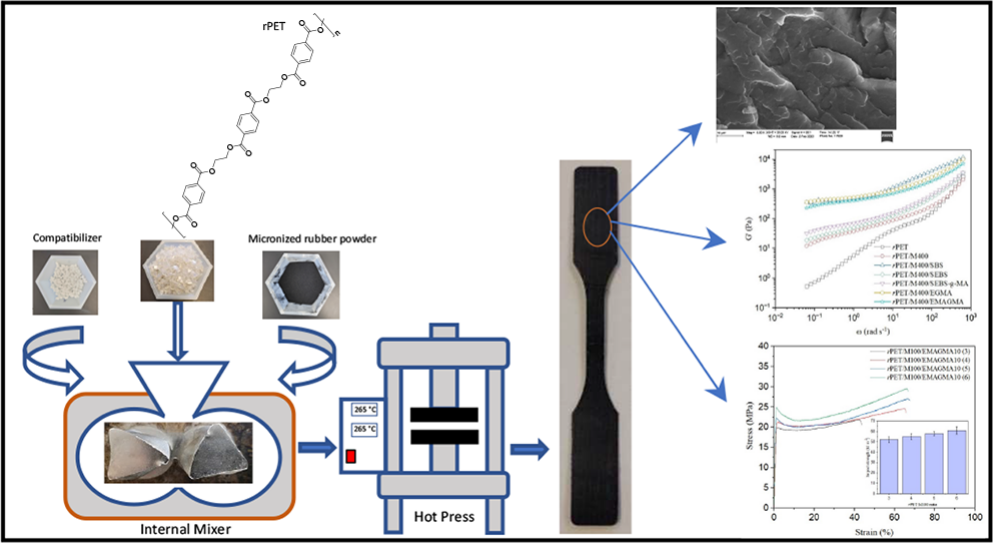

Plastic pollution has become a pressing global crisis that threatens biodiversity and reduces the adaptability of the ecosystem to climate change. Additive manufacturing technologies hold promise in the context of distributed recycling and sustainability. The present work elaborates on developing low-cost, robust feedstocks with improved toughness based on postconsumer polyethylene terephthalate, *r*PET, and micronized scrap tire rubber powder (MRP) for additive manufacturing. The effects of a series of nonreactive (polystyrene-*block*-polybutadiene-*block*-polystyrene (SBS) and polystyrene-*block*-poly(ethylene-*ran*-butylene)-*block*-polystyrene (SEBS)) and reactive compatibilizers (polystyrene-*block*-poly(ethylene-*ran*-butylene)-*block*-polystyrene-*g*-maleic anhydride (SEBS-*g*-MA), poly(ethylene-*co*-glycidyl methacrylate) (EGMA), and poly(ethylene-*co*-methyl acrylate-*co*-glycidyl methacrylate) (EMAGMA)) on the mechanical and rheological properties of *r*PET/MRP composites were investigated. *r*PET/MRP composites comprising compatibilizers with glycidyl moieties showed relatively higher impact strength and elongation at break. Rheological measurements revealed that incorporating MRP into *r*PET in the presence of compatibilizers remarkably increases melt viscosity, making the composite formulation suitable for extrusion processing. Differential scanning calorimetry results disclosed that reactive compatibilizers favorably reduce composite crystallinity compared to non-reactive ones, which are ascribed to the formation of long-chain branches. The potential of *r*PET/MRP filaments for fused deposition modeling was screened by using a low-budget desktop 3D printer. It is envisioned that the findings of this study will improve resource efficiency and the supply chain to achieve a waste-free economy and sustainability.

## Introduction

Plastic production, consumption, and disposal are growing relentlessly and have caused a severe environmental crisis.^1−3^ Plastic waste generation is forecasted to reach 12 billion tons worldwide by 2050.^4^ Macroscale plastic pieces and microplastics have significant adverse impacts on human health and the ecosystem. Over the past years, the shift from reusable to single-use containers has accelerated the growth of plastic packaging.^5^ The excellent mechanical properties, high chemical resistance, and stability over a wide range of temperatures (−60 to 220 °C) have made polyethylene terephthalate (PET) a material of choice for disposable bottles and food packaging.^6,7^ Approximately more than 580 billion plastic bottles were produced in 2021, of which about 60% were made of PET. Despite being highly recyclable, less than 10% of PET bottles were recycled in 2016.^8^

Nonstop expansion of the automotive industry requires the production of 1.5 billion tires annually worldwide.^9^ Approximately one billion tires reach their end-of-life each year, necessitating environmentally safe and cost-effective approaches for their disposal.^10,11^ However, there have yet to be satisfactory solutions that can be applied on an industrially practical scale.^12,13^

Scrap tires can be used as a supplement to conventional fuels, but upon burning, they generate uncontrollable side products. To address this challenge, alternative grinding technologies have been demonstrated to be effective at producing micronized tire rubber powder (MRP), which can be mixed with other compounds (e.g., thermoplastics, bitumen).^14−17^ Thermoplastic/MRP composites are attractive because of enhanced mechanical properties, ease of processing, and low cost.^18,19^ Incorporation of an elastomeric phase into thermoplastic matrices offers improved impact behavior.^20,21^ To increase fracture toughness, dispersed MRP particles need to fulfill two functions: concentrate local stress and debond to change the stress state of the matrix, leading to overall matrix deformation.^22,23^ The propagating crack is blunted by coalesced voids, providing a deviation of the crack path around the void. It has been shown that mechanical performance (i.e., elongation at break, impact strength) is strongly influenced by grain size, the content of MRP particles, and the interfacial bonding to the matrix (i.e., using compatibilizers).^24^

Butadiene and styrene-butadiene rubber stand as the primary synthetic polymers (constituting around 18–27% by weight) used in tire production, typically blended with natural rubber (approximately 14–23% by weight).^25^ MRP has a solubility parameter (δ) of ∼17 MPa^1/2^ compared to that of 22 MPa^1/2^ for PET as a polar polymer, implying their immiscibility.^26,27^ To address this issue, surface modification of MRP by grafting functional groups such as peroxyl,^28^ hydroxyl,^29^ and carbonyl^30^ has been studied. Furthermore, other modification methods, including UV,^31^ electron beam,^32^ and γ-ray^33^ radiation, as well as adding suitable compatibilizers,^34−36^ have been introduced to achieve better interfacial adhesion. Herein, we explore the efficiency of nonreactive and reactive compatibilizers (i.e., copolymers) to improve PET/MRP miscibility. Compatibilizers capable of interacting with polymers in an immiscible blend can induce partial miscibility by forming bridges among phases.^37^ Hence, the interfacial adhesion is increased, which results in an improvement of the mechanical properties in the composites. Typically, compatibilizers decrease the size of the dispersed phase and offer enhanced morphological stability.^38^ There are several reports on using nonreactive compatibilizers to reduce the immiscibility of MRP in polymer composites. Qin et al. stated that the combination of linear low-density polyethylene (LLDPE) and MRP, which includes SBS copolymer as a compatibilizer, demonstrated commendable mechanical properties, even when the MRP content was relatively high (around 60%).^39^ Atomic force microscopy (AFM) and differential scanning calorimetry (DSC) revealed improved adhesion between rubber particles and the LLDPE matrix. Rocha et al. demonstrated an enhancement in the tensile strength of LLDPE/MRP composites utilizing ethylene-1-octene metallocene copolymer as a nonreactive compatibilizer.^40^ Wang et al. documented that the inclusion of compatibilizers in recycled polyethylene (*r*PE) and MRP composites resulted in improved mechanical properties. Specifically, composites containing ethylene-octene copolymer showcased higher tensile strength and elongation at break. Interestingly, the impact strength remained largely unaffected by the MRP content.^41^ In another study, Fazli and Rodrigue examined thermoplastic elastomers (TPEs) comprising recycled high-density polyethylene (*r*HDPE), MRP, and ethylene vinyl acetate (EVA) utilized as a compatibilizer.^42^ The results revealed that partial substitution of *r*HDPE by EVA (5–15 *wt*%) enhances TPE homogeneity and elongation at break.

Weak interaction between the phases in MRP composites often originates from the cross-linked structure and the low affinity of rubber molecules to entangle with the thermoplastic matrix chains. Reactive compounding techniques to improve the compatibility of immiscible polymers require compatibilizers (coupling agents) with functional groups such as maleic anhydride,^43^ isocyanate,^44^ epoxide,^45^ and oxazoline.^46^ Reactive blending typically results in complex structures with the coupling agent at droplet interfaces, thus achieving a finer and more uniform morphology and ultimately improving mechanical properties.^47^ A dual reactive compatibilizer based on LLDPE grafted with maleic anhydride, methyl methacrylate, butyl acrylate, and epoxidized natural rubber has been investigated for TPEs derived from scrap rubber powder/LLDPE composites.^48^ The TPEs comprising a compatibilizer showed significantly improved mechanical properties, particularly elongation at break. Maleated polyethylene (MAPE, maleic anhydride grafted polyethylene, PE-*g*-MA) has been used both as the matrix and reactive compatibilizer to formulate highly MRP-filled TPEs.^49^ While MAPE/MRP compounds comprising 50–70% MRP demonstrated high elongation at break (>400%), tensile properties were found to decrease at higher MRP content. In another study, the effects of MAPE as a reactive compatibilizer on the mechanical and swelling behavior of HDPE/EVA/MRP composites were investigated.^50^ It was found that increasing MRP content causes a dramatic decrease in the tensile strength of composites, while PE-*g*-MA enhanced the compatibility of phases and resulted in higher tensile strength. Although compatibilization enhances the interfacial adhesion and mechanical performance of HDPE/EVA/MRP composites, the overall extent of chain modification remains relatively low. This limited modification allows for further design and optimization of material properties, as discussed in previous studies.^51^ The study investigated the rubber toughening of *r*PET using micronized rubber powder and nonreactive and functionalized elastomers based on SEBS. Analysis of the fracture surface revealed evidence of ductile fracture and cavitation in the rubber-toughened samples. The results showed that increasing elastomers did not have a significant effect on the tensile strength, but the toughness increased up to 550% with SEBS-*g*-MA compared to the neat *r*PET.^22^

The preceding discussion highlights the increasing need for and initiatives for recycling of waste plastics and tires. At the same time, there has been a surge in additive manufacturing (AM), which has created avenues for more research aimed at producing innovative and affordable 3D printable polymers. The assessment of degradation mechanisms, such as thermal and thermo-mechanical processes during AM and recycling, is critical to ensuring both the performance of the final material and its alignment with material circularity goals.^52−59^

Rubber-toughened recycled materials are becoming increasingly popular. Zander and Boelter developed tough filaments for material extrusion additive manufacturing using rubber powder and elastomers. Their study focused on investigating the mechanical properties of the rubberized *r*PET, including its ultimate tensile strength, toughness, and modulus of elasticity.^22^ Selective laser sintering was used as a 3D printing technique to incorporate recycled MRP into polyamide and thermoplastic polyurethane matrices. The mechanical properties and thermal stability of the samples were also studied.^60^

The main objective of this work is to improve the impact resistance and energy absorption of *r*PET that is useful for applications requiring flexibility and toughness. In addition, enhancing the melt rheological properties and viscosity of *r*PET by adding MRP can help to create a cost-effective, robust 3D printable feedstock. Cost-effective, recycle-based MRP-toughened objects were produced using a desktop, inexpensive fused filament fabrication (FFF) 3D printer, which is a simpler and more affordable approach compared to complex custom-made systems used in previous studies. The use of low-cost printing systems can greatly enhance the commercialization of additively manufactured materials from recycled waste tires and plastics. However, it is important to note that the FFF process introduces an additional thermal degradation step, which may influence the mechanical and rheological properties of the recycled polymer-based composites. Therefore, optimizing processing conditions and incorporating appropriate stabilizers are critical considerations for maintaining performance.

**Scheme 1:**
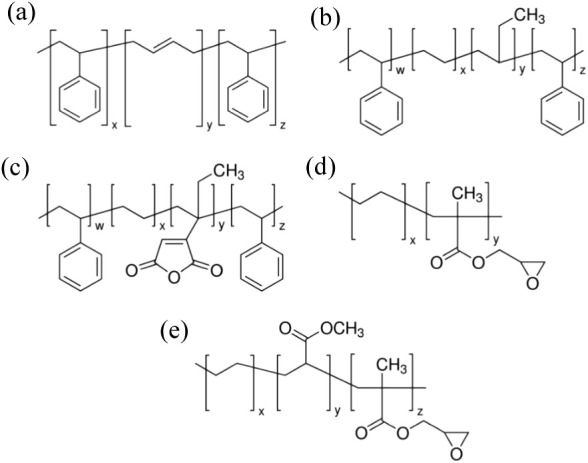
Chemical Structure of Compatibilizers (a) Polystyrene-*block*-polybutadiene-*block*-polystyrene (SBS), (b) polystyrene-*block*-poly(ethylene-*ran*-butylene)-*block*-polystyrene (SEBS), (c) polystyrene-*block*-poly(ethylene-*ran*-butylene)-*block*-polystyrene-*g*-maleic anhydride (SEBS-*g*-MA), (d) poly(ethylene-*co*-glycidyl methacrylate) (EGMA), and (e) poly (ethylene-*co*-methyl acrylate-*co*-glycidyl methacrylate) (EMAGMA)

We comprehensively investigated the effects of different types of reactive and nonreactive compatibilizers on the mechanical, thermal, rheological, and morphological properties of *r*PET/MRP immiscible composites. SBS and SEBS were used as nonreactive compatibilizers. The reactive coupling agents utilized were SEBS-*g*-MA, EGMA, and EMAGMA (Scheme 1).

## Results and Discussion

**Figure 1:**
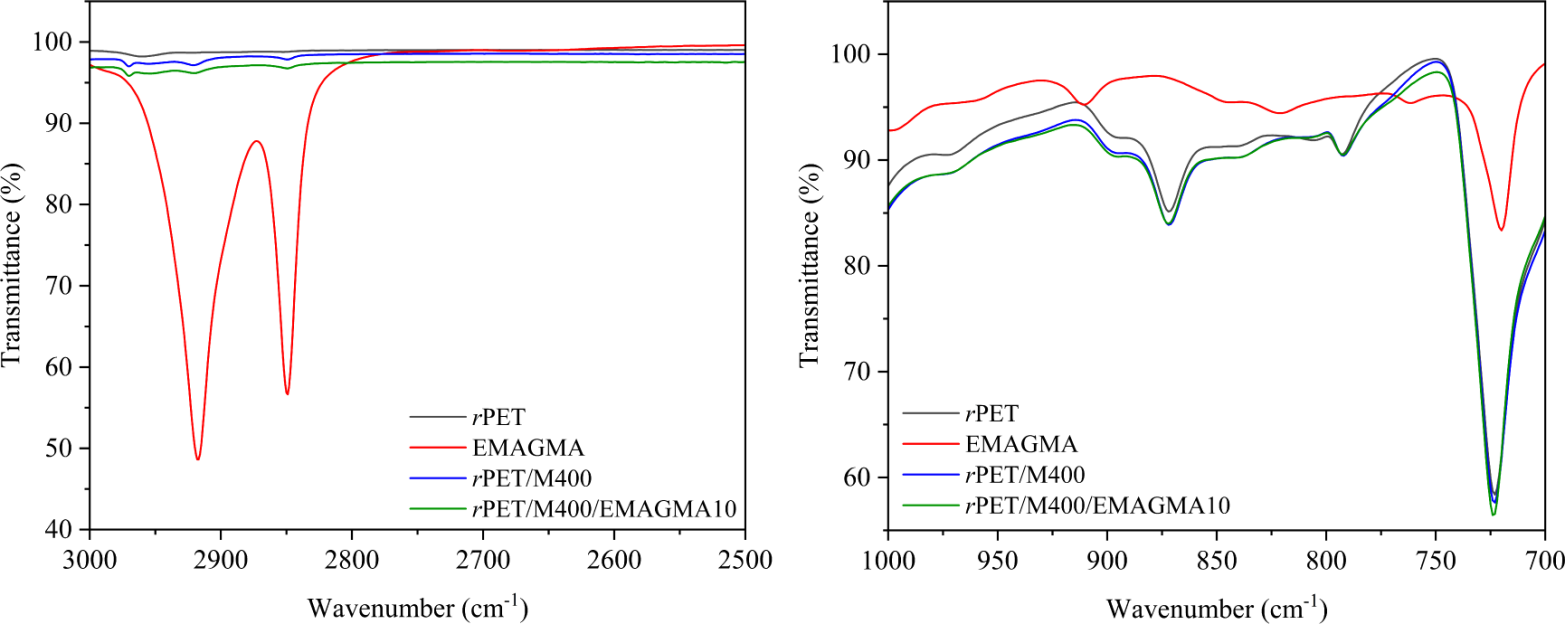
Fourier-transform infrared spectra of compatibilized *r*PET/MRP composites in comparison with neat *r*PET, uncompatibilized blend, and EMAGMA as a compatibilizer over different wavenumber ranges.

To examine the interactions of compatibilizers with the matrix, *r*PET/MRP composites were characterized by Fourier transform infrared (FTIR) spectroscopy. FTIR spectra of *r*PET, EMAGMA, uncompatibilized *r*PET/MRP, and *r*PET/MRP with EMAGMA composites are shown in Figure 1. In the EMAGMA spectrum, peaks at 911 and 844 cm^–1^ are attributed to the C–O–C stretching vibrations of the epoxide groups. Also, the symmetrical and asymmetrical stretching vibration absorption peaks of the methyl group are located at 2920 and 2850 cm^–1^.^61^ The disappearance of these peaks in the EMAGMA-compatible *r*PET/MRP blend spectrum demonstrates the consumption of all epoxide groups of EMAGMA during the chemical reaction with *r*PET. Thus, it can be concluded that branched structures were formed. FTIR spectra of the SBS-compatibilized *r*PET/MRP blend compared with neat *r*PET, SBS and the uncompatibilized *r*PET/MRP blend are shown in Figure S1. The peaks at 754 and 698 cm^–1^ are ascribed to monosubstituted benzene rings of polystyrene blocks of SBS. The strong peak at 964 cm^–1^ originates from the dominant trans-1,4-butadiene structure, while the weak peak at 910 cm^–1^ is derived from the minor 1,2-butadiene of the polybutadiene block.^62^ For SEBS, the band at 1601–1452 cm^–1^ is assigned to the skeleton vibration of the benzene rings (Figure S2). A monosubstituted benzene ring can be attributed to the peaks at 758 cm^–1^ and 697 cm^–1^. The peak at 1378 cm^–1^ corresponds to the C–H bending vibration of the ethylene-butylene segments.^63^ FTIR spectra in Figures S1 and S2 confirm that SBS and SEBS were blended with the *r*PET/MRP composites. Figure S3 demonstrates FTIR spectra of the compatibilized *r*PET/MRP blend comprising SEBS-*g*-MA compared with neat *r*PET, SEBS-*g*-MA, and the corresponding uncompatibilized blend. Generally, maleic anhydride shows two peaks at 1871 and 1793 cm^–1^ for asymmetric and symmetric vibrations of C=O groups, respectively. Although SEBS-*g*-MA did not exhibit peaks at the aforementioned wavenumbers, the observed band at 1715 cm^–1^ implies C=O dimers present in COOH dimers.^64^ Peaks at 1602 and 1584 cm^–1^ are typical wavenumber positions for aromatic ring stretching vibrations. EMAGMA and EGMA contain epoxide, which can react with the hydroxyl and carboxyl groups of *r*PET chains to form branched structures. Figure S4 shows the FTIR spectra of *r*PET, EGMA, uncompatibilized *r*PET/MRP, and compatibilized *r*PET/MRP with EGMA samples. The spectrum of pure EGMA presents characteristic peaks at 997, 911, and 847 cm^–1^, which are characteristics of epoxy groups in EGMA.^65^ As for *r*PET/MRP composites compatibilized with EGMA, the epoxide bands at 945 and 911 cm^–1^ disappear after blending, which indicates that an epoxy ring-opening reaction may occur. This could be attributed to the aforementioned reaction between the epoxy groups on EGMA and the terminal carboxylic acid or terminal hydroxyl group on *r*PET involved in this compatibilized blend. Compared to uncompatibilized blends, our compatibilized composites exhibited more pronounced peak shifts and intensities, confirming stronger polymer–filler interactions. Prior studies on *r*PET-based blends have reported limited interfacial adhesion without compatibilizers, often leading to phase separation and inferior mechanical properties.^66,67^

**Figure 2:**
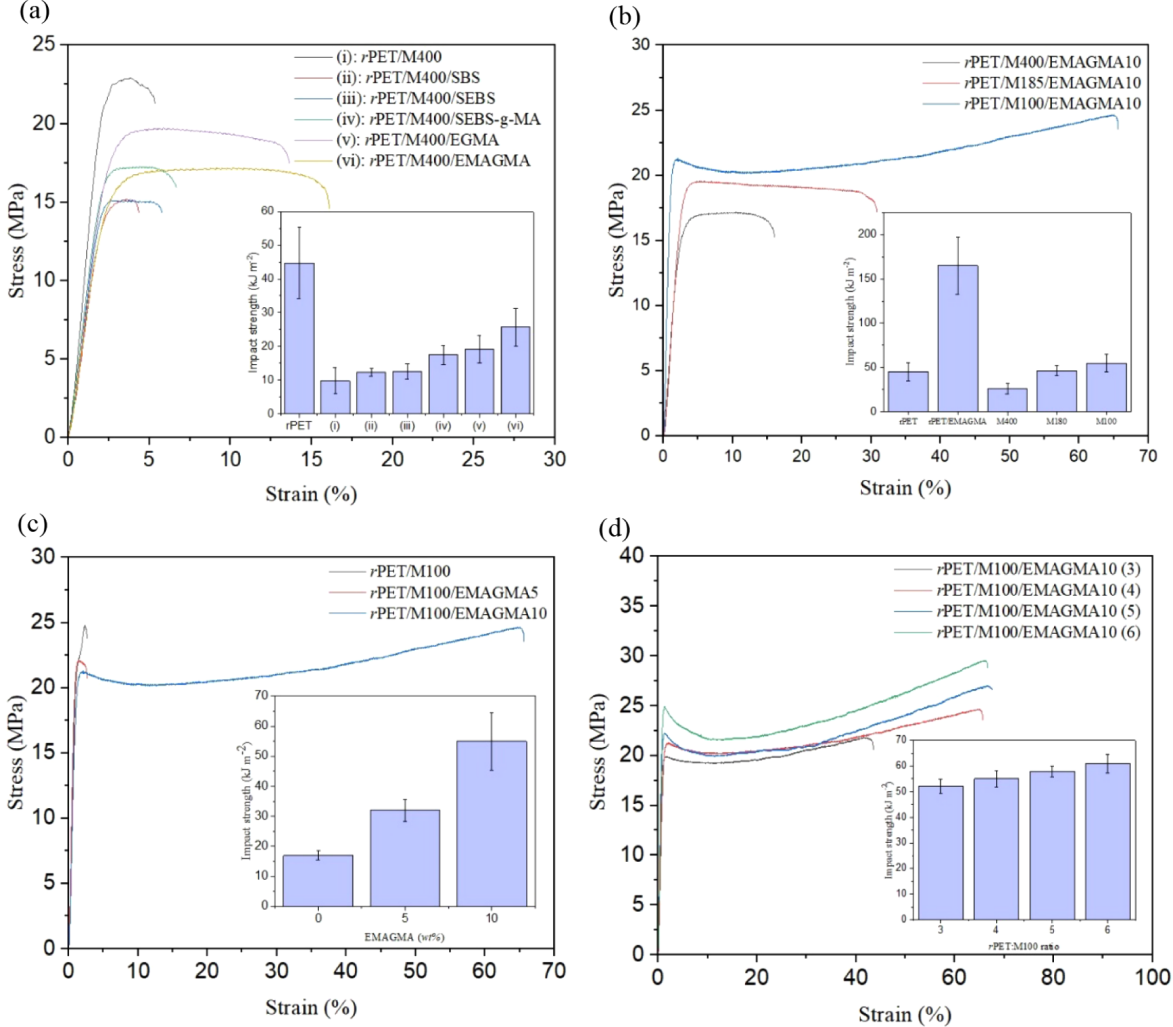
Stress–strain curves and impact strength (inset graphs) of *r*PET/MRP composites: (a) compatibilized using different compatibilizers (SBS, SEBS, SEBS-*g*-MA, EGMA, and EMAGMA) at 10 *wt*% in comparison with the uncompatibilized *r*PET/MRP blend (MRP particle size 400 μm) and the neat *r*PET. (b) Effect of MRP particle sizes of 100, 185, and 400 μm at EMAGMA 10 *wt*%. (c) Effect of EMAGMA content at 5 and 10 *wt*% and MRP particle size of 100 μm. (d) Effect of rPET/MRP ratio at EMAGMA 10 *wt*% and MRP particle size of 100 μm (see Table 3 for sample composition).

**Scheme 2:**
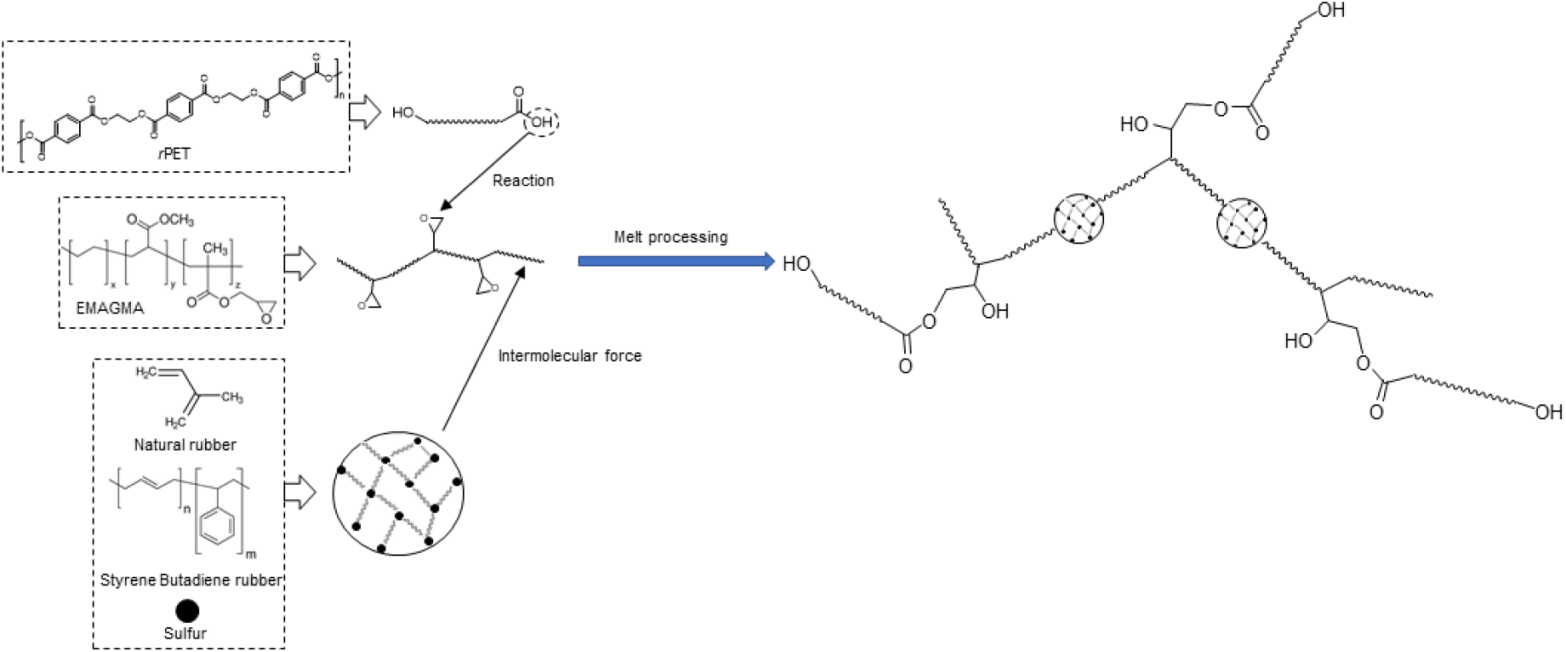
Possible Reaction Routes and Intermolecular Forces in *r*PET/MRP Composite with the Inclusion of EMAGMA

Stress–strain curves of *r*PET/MRP compounds compatibilized using different compatibilizers (SBS, SEBS, SEBS-*g*-MA, EGMA, and EMAGMA), in comparison with the uncompatibilized *r*PET/MRP blend, are demonstrated in Figure 2. PET is a ductile polymer with a Young’s modulus (YM) of 1900 MPa, a tensile strength (TS) of 50 MPa, and an elongation at break (EB) of ∼400%. After melt blending *r*PET with 20 *wt*% MRP, a significant decrease in YM and TS was observed, with respective values of 1119 and 20.3 MPa for the uncompatibilized blend. Further, EB dropped to 6% after the incorporation of MRP. As shown in Figure 2a, the addition of 10 *wt*% reactive compatibilizers resulted in an improvement of EB to 18%, 15%, and 8% when using EMAGMA, EGMA, and SEBS-*g*-MA as compatibilizers, respectively. The mechanical properties of the *r*PET/MRP compounds are summarized in Table 1. The role of EMAGMA in achieving a higher EB compared to other compatibilizers can be interpreted through its chemical structure (Scheme 1). The enhanced compatibilization performance of EMAGMA can be attributed to the presence of methyl acrylate (MA) segments in addition to glycidyl methacrylate (GMA) and polyethylene (PE) blocks. PE domains of EMAGMA have a high chemical affinity with MRP, while the epoxide (GMA) blocks of EMAGMA react with *r*PET (Scheme 2). The MA segments introduce greater flexibility and polarity to EMAGMA compared to EGMA. This allows the EMAGMA triblock copolymer to act as a bridge between the two immiscible phases, hence improving their compatibility and interfacial adhesion and reducing the stress concentration effect caused by the MRP-rich domains, consistent with previous findings in compatibilized PET-based blends.^37^ The lack of extra polarity from the MA segments in EGMA, despite having GMA blocks, may reduce its compatibilization effectiveness. EB of *r*PET/MRP composites was found to be insignificantly affected by nonreactive compatibilizers (i.e., SBS and SEBS).

**Table 1:** Summarized Mechanical Properties of *r*PET/MRP Composites and Neat *r*PET (see Table 3 for sample composition)

Sample	Tensile Strength (MPa)	Young’s Modulus (MPa)	Elongation at Break (%)	Impact Energy (kJ m^–2^)
*r*PET	50.1 ± 8.4	1901 ± 67	396.3 ± 45.3	44.7 ± 10.5
*r*PET/M400	20.3 ± 2.5	1119 ± 50	5.4 ± 1.7	9.8 ± 3.9
*r*PET/M100	25.1 ± 2.2	1235 ± 28	5.3 ± 2.0	17.1 ± 1.5
*r*PET/M400/SBS	16.5 ± 1.7	841 ± 30	4.3 ± 1.1	12.4 ± 1.2
*r*PET/M400/SEBS	15.1 ± 3.0	895 ± 31	5.8 ± 1.8	12.6 ± 2.3
*r*PET/M400/SEBS-*g*-MA	14.8 ± 3.9	787 ± 28	6.7 ± 2.1	17.5 ± 2.8
*r*PET/M400/EGMA	19.7 ± 3.9	888 ± 35	13.7 ± 2.1	19.2 ±4.1
*r*PET/M400/EMAGMA	17.2 ± 4.3	788 ± 32	16.1 ± 3.2	25.7± 5.7
*r*PET/M185/EMAGMA	20.7 ± 3.3	835 ± 36	30.9 ± 6.0	46.3 ± 5.7
*r*PET/M100/EMAGMA5	22.5 ± 3.5	1050 ± 22	7.1 ± 2.1	31.6 ± 3.7
*r*PET/M100/EMAGMA10	22.7 ± 3.1	915 ± 34	53.2 ± 6.1	55.5 ± 9.6
*r*PET/M100/EMAGMA10 (3)	21.8 ± 1.9	1052 ± 29	43.6 ± 6.3	52.1 ± 2.9
*r*PET/M100/EMAGMA10 (4)	22.7 ± 3.1	915 ± 34	53.2 ± 6.1	55.5 ± 3.2
*r*PET/M100/EMAGMA10 (5)	23.2 ± 1.1	890 ± 12	54.1 ± 6.7	58.2 ±2.2
*r*PET/M100/EMAGMA10 (6)	26.1 ± 3.7	866 ± 17	55.1 ± 6.2	61.4 ± 3.7
*r*PET/EMAGMA	47.7 ± 1.1	1301 ± 61	347.5 ± 29.1	165.2 ± 32.5

As an indicator of toughness, the impact-absorbed energy for *r*PET/MRP compounds compatibilized using different compatibilizers (SBS, SEBS, SEBS-*g*-MA, EGMA, and EMAGMA) was compared with that of the uncompatibilized *r*PET/MRP blend (please see the inset graphs in Figure 2). Absorption capacity depends notably on the material cohesion, which is one of the most sensitive properties to the compatibility between polymers in a blend.^68^ The observed increase in energy absorption capacity of the blend after the incorporation of compatibilizers (reactive and nonreactive) used in this study suggests an improvement in the interaction between both phases and, therefore, an increase in blend continuity. The modified Charpy’s impact energy value for neat *r*PET was 44.7 kJ m^–2^, indicating a low to medium energy absorption. The blend of *r*PET with MRP resulted in a significant decrease in the energy absorption capacity compared to neat *r*PET, leading to an energy absorption value in the *r*PET/MRP blend of 9.8 kJ m^–2^, representing a decrease of 356% compared to neat *r*PET. In the same way as elongation at break, phase separation occurs due to low interfacial adhesion between both polymers. Stress concentration occurs due to this lack of interaction among the interfaces, which makes the material more brittle. As shown in the inset graphs in Figure 2a, the impact-absorbed energy increased after the addition of the different compatibilizers. As a result, the EMAGMA-compatibilized blend at 10 *wt*% content showed the highest energy absorption, reaching 25.7 kJ m^–2^, representing a 161% increase over the uncompatibilized blend (MRP particle size of 400 μm). Figure 2b demonstrates the effect of MRP particle size (400, 180, and 100 μm) on stress–strain behavior and impact strength of the *r*PET/MRP composites comprising EMAGMA (10 *wt*%) as a reactive compatibilizer. By reducing the MRP particle size, mechanical properties in terms of YM, EB, TS, and impact strengths were remarkably improved (Table 1). This finding could be explained by a more uniform dispersion of MRP of smaller size, which resulted in a reduction of the stress concentration effects in the *r*PET matrix. The effect of the EMAGMA content (5 and 10 *wt*%) on the mechanical properties of *r*PET blended with MRP particles of 10 μm is displayed in Figure 2c. It was found that increasing EMAGMA content (within the loading range of compatibilizers in Table 3) in *r*PET/MRP composites leads to a drastic increase in impact strength (i.e., *r*PET/M100/EMAGMA10 offers 25% higher impact strength than the neat *r*PET) and EB but decreased YM and TS (Table 1). The effect of the *r*PET-to-MRP ratio (*r*PET:MRP) on the mechanical properties of composites comprising 10 *wt*% EMAGMA is demonstrated in Figure 2d. With an increasing *r*PET:M100 ratio, mechanical properties (i.e., YM, EB, TS, and impact strengths) were improved. This observation is due to a better distribution of MRP at lower content in the *r*PET matrix, which resulted in better load transfer along the interface and effective energy absorption. The mechanical properties of *r*PET/MRP composites are summarized in Table 1 (sample compositions are tabulated in Table 3).

**Figure 3:**
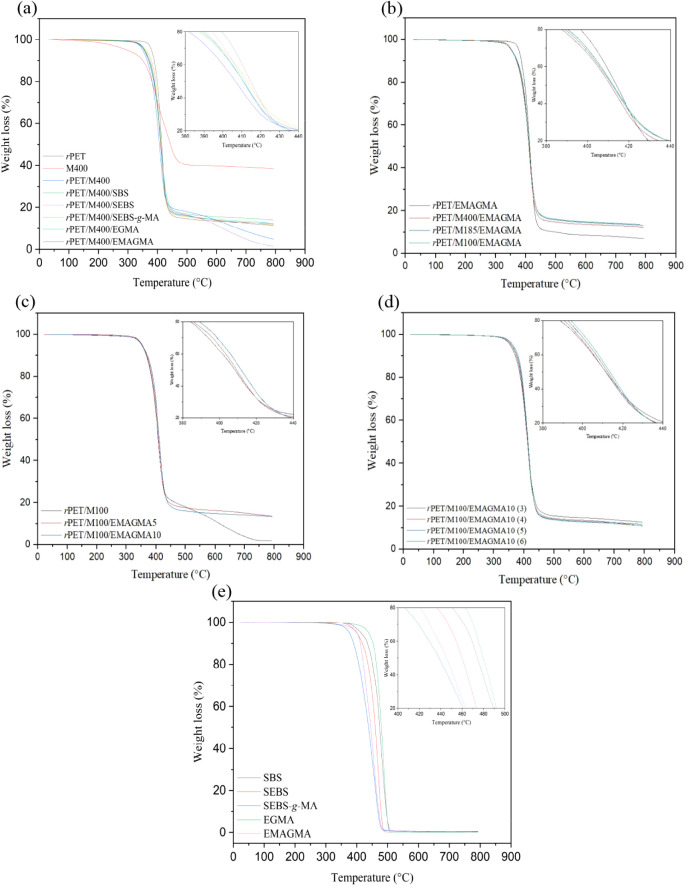
Thermogravimetric curves of (a) compatibilized *r*PET/MRP composites using different compatibilizers (SBS, SEBS, SEBS-*g*-MA, EGMA, and EMAGMA) at 10 *wt*% in comparison with the uncompatibilized *r*PET/MRP blend (MRP particle size 400 μm), and the neat *r*PET. (b) Effect of MRP particle sizes of 100, 185, and 400 μm at EMAGMA 10 *wt*%. (c) Effect of EMAGMA content at 5, and 10 *wt*% and MRP particle size of 100 μm. (d) Effect of *r*PET/MRP ratio at EMAGMA 10 *wt*% and MRP particle size of 100 μm. (e) Reactive and nonreactive compatibilizers (see Table 3 for sample composition).

Thermogravimetric analysis (TGA) was performed on *r*PET/MRP composites to determine their thermal stability. It was found that the incorporation of MRP into the *r*PET matrix decreases the onset degradation temperature and the overall thermal stability of the blend. The temperature at the 5% weight loss in the decomposition stage (T_5%_) for uncompatibilized *r*PET/MRP was measured to be ∼350 °C, while it was found to be ∼377 °C for neat *r*PET (Figure 3a).

Compatibilized *r*PET/MRP composites showed T_5%_ values slightly higher than those of the uncompatibilized compounds. While T_5%_ was found to marginally increase with a smaller MRP size for the uncompatibilized *r*PET/MRP composites, no significant particle size effect was observed for the *r*PET/MRP/EMAGMA composites (Figure 3b). This observation implies the effectiveness of the reactive compatibilizer in achieving uniform dispersion of MRP in the *r*PET matrix. Figure 3c demonstrates that with increasing EMAGMA content, the thermal stability of compatibilized *r*PET/MRP composites is hardly affected.

Such an observation infers uniform dispersion of MRPs even at a low compatibilizer content of 5 *wt*%. Generally, the formation of long-chain branches (LCBs) in the presence of reactive compatibilizers increases the onset degradation temperature due to an increase in the number of entanglements per chain, but the entanglement density may reach a plateau value. This behavior has been previously observed in chemically modified PET-based blends.^69^ EMAGMA-compatibilized composites with a higher ratio of *r*PET:MRP showed higher T_5%_, which is consistent with the adverse effect of the incorporation of MRP into the *r*PET matrix (Figure 3d). The thermal stability results of the *r*PET/MRP composites are summarized in Table 2 (see Table 3 for composite compositions).

**Table 2:** Summarized Thermal Properties^a^ Obtained from Differential Scanning Calorimetry and Thermogravimetric Characterization of *r*PET/MRP Composites and the Neat *r*PET (See Table 3 for Sample Composition)

Sample	*T*_g_ (°C)	*T*_c_ (°C)	*T*_m_ (°C)	*ΔH*_m_ (J g^–1^)	*ΔH*_c_ (J g^–1^)	*X*_c_ (%)	*T*_5%_ (°C)
*r*PET	73.6 ± 0.2	206.2 ± 0.5	248.5 ± 0.2	43.4 ± 0.5	53.8 ± 0.4	35 ± 0.1	379.9 ± 1.2
*r*PET/M400	71.7 ± 0.1	215.4 ± 0.3	248.4 ± 0.1	41.3 ± 0.5	42.1 ± 0.4	42 ± 0.3	350.2 ± 1.5
*r*PET/M100	69.8 ± 0.2	213.5 ± 0.3	244.7 ± 0.2	41.6 ± 0.4	41.9 ± 0.4	43 ± 0.2	352.1 ± 1.9
*r*PET/M400/SBS	70.5 ± 0.3	212.9 ± 0.2	247.8 ± 0.1	36.4 ± 0.5	37.9 ± 0.4	41 ± 0.3	357.1 ± 1.9
*r*PET/M400/SEBS	69.9 ± 0.1	213.5 ± 0.1	247.1 ± 0.2	38.3 ± 0.3	39.1 ± 0.4	44 ± 0.3	355.6 ± 2.0
*r*PET/M400/SEBS-*g*-MA	71.1 ± 0.1	211.3 ± 0.1	247.7 ± 0.2	38.3 ± 0.4	39.3 ± 0.4	43 ± 0.3	360.9 ± 2.0
*r*PET/M400/EGMA	70.6 ± 0.1	211.3 ± 0.1	247.8 ± 0.2	31.1 ± 0.4	32.7 ± 0.4	35 ± 0.1	356.2 ± 1.7
*r*PET/M400/EMAGMA	73.3 ± 0.2	211.2 ± 0.1	247.4 ± 0.2	34.3 ± 0.4	34.2 ± 0.5	39 ± 0.2	354.9 ±1.7
*r*PET/M185/EMAGMA	71.9 ± 0.1	211.4 ± 0.2	245.9 ± 0.2	36.9 ± 0.3	36.9 ± 0.4	42 ± 0.2	354.3±1.5
*r*PET/M100/EMAGMA5	73.6 ± 0.1	213.6 ± 0.1	247.7 ± 0.1	38.9 ± 0.4	40.2 ± 0.4	42 ± 0.2	354.2±1.1
*r*PET/M100/EMAGMA10	71.8 ± 0.1	211.6 ± 0.2	247.3 ± 0.2	37.2 ± 0.4	37.1 ± 0.4	42 ± 0.2	355.1 ± 1.8
*r*PET/M100/EMAGMA10 (3)	70.3 ± 0.2	212.3 ± 0.1	246.9 ± 0.1	36.8 ± 0.5	36.5 ± 0.5	44 ±0.2	354.5 ± 2.2
*r*PET/M100/EMAGMA10 (4)	71.8 ± 0.2	211 .6 ± 0.3	247.3 ± 0.2	37.2 ± 0.3	37.1 ± 0.4	42 ± 0.3	355.1 ± 1.5
*r*PET/M100/EMAGMA10 (5)	72.6 ± 0.2	208.9 ± 0.1	249.6 ± 0.1	38.1 ±0.3	38.0 ±0.5	41 ± 0.1	360.5 ± 1.6
*r*PET/M100/EMAGMA10 (6)	73.3 ± 0.1	208.5 ± 0.1	249.9 ± 0.2	40.3 ± 0.4	40.1 ± 0.3	41 ± 0.1	364.3 ± 1.4
*r*PET/EMAGMA	71.7 ± 0.2	212.1 ± 0.2	249.6 ± 0.2	44.1 ± 0.5	43.9 ± 0.4	40 ± 0.2	377.9 ± 1.8

a *T*_g_: glass transition temperature, *T*_c_: crystallization temperature, *T*_m_: melting temperature, *ΔH*_m_: melting enthalpy, *ΔH*_c_: crystallization enthalpy, *X*_c_: degree of crystallinity, *T*_5%_: temperature at 5% weight loss in decomposition stage.

**Table 3:** Formulation of the *r*PET/MRP compounds

Sample	*r*PET (*wt*%)	Compatibilizer	Compatibilizer (*wt*%)	MRP Particle size (um)	MRP (*wt*%)
*r*PET	100	--	0.00	--	0.00
*r*PET/M400	80.00	--	0.00	400	20.00
*r*PET/M100	80.00	--	0.00	100	20.00
*r*PET/M400/SBS	72.00	SBS	10.00	400	18.00
*r*PET/M400/SEBS	72.00	SEBS	10.00	400	18.00
*r*PET/M400/SEBS-*g*-MA	72.00	SEBS-*g*-MA	10.00	400	18.00
*r*PET/M400/EGMA	72.00	EGMA	10.00	400	18.00
*r*PET/M400/EMAGMA	72.00	EMAGMA	10.00	400	18.00
*r*PET/M185/EMAGMA	72.00	EMAGMA	10.00	185	18.00
*r*PET/M100/EMAGMA5	76.00	EMAGMA	5.00	100	19.00
*r*PET/M100/EMAGMA10	72.00	EMAGMA	10.00	100	18.00
*r*PET/M100/EMAGMA10 (3)	67.5	EMAGMA	10.00	100	22.50
*r*PET/M100/EMAGMA10 (4)	72.00	EMAGMA	10.00	100	18.00
*r*PET/M100/EMAGMA10 (5)	75.00	EMAGMA	10.00	100	15.00
*r*PET/M100/EMAGMA10 (6)	77.14	EMAGMA	10.00	100	12.86
*r*PET/EMAGMA	90.00	EMAGMA	10.00	--	0.00

**Figure 4:**
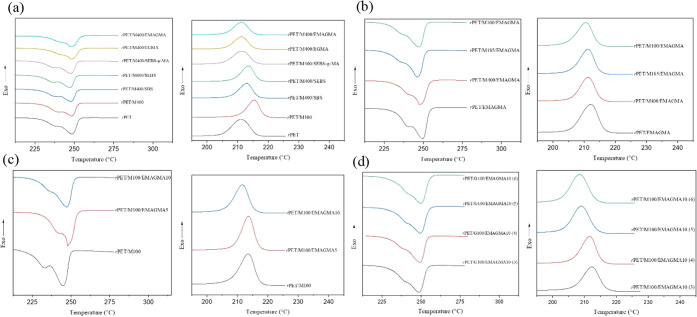
Differential scanning calorimetry thermograms of *r*PET/MRP composites over the heating and cooling cycles: (a) effect of different compatibilizers (SBS, SEBS, SEBS-*g*-MA, EGMA, and EMAGMA) at 10 *wt*% in comparison with the uncompatibilized *r*PET/MRP blend (MRP particle size 400 μm) and the neat *r*PET. (b) Effect of MRP particle sizes of 100, 185, and 400 μm at EMAGMA 10 *wt*%. (c) Effect of EMAGMA content at 5 and 10 *wt*% and MRP particle size of 100 μm. (d) Effect of *r*PET/MRP ratio at EMAGMA 10 *wt*% and MRP particle size of 100 μm (see Table 3 for sample composition).

Thermal properties of the *r*PET/MRP composites were further characterized through differential scanning calorimetry (DSC) over the heating and cooling cycles. It should be noted that the second heating thermogram is plotted (Figure 4a–-d). It was found that the incorporation of MRP into the *r*PET matrix decreases its glass transition temperature (*T*_g_). This effect becomes more pronounced with decreasing particle size and after the addition of the nonreactive compatibilizers, which originates from their rubbery nature. In the case of reactive compatibilizers, the formation of branched structures can result in a lower *T*_g_ of the polymer due to increased free volume.^70^ Due to the increased branch density, side chains disrupt the packing of polymer chains, the hindrance to segmental mobility is reduced, and consequently, *T*_g_ is lowered.^71^ However, when the branch density reaches a critical value, *T*_g_ no longer drops and reverses.^72^ In this view, rising *T*_g_ with an increasing *r*PET:MRP ratio stemmed from the formation of more branched structures and chain entanglements in the EMAGMA-compatibilized composites. Moreover, the formation of branches in the compatibilized composites caused the melting temperature (*T*_m_) and enthalpies to decrease, which implies impeded formation of the crystalline phase.^73^ One can define the degree of crystallinity (*X*_c_) by normalizing the observed melting temperature to that of a fully crystalline polymer: *X*_c_ = *ΔH*_m_/*ΔH*_m_*, where *ΔH*_m_ is the melting enthalpy and *ΔH*_m_* is the melting enthalpy of a fully crystalline polymer, which equals 121 J g^–1^ for PET.^74^ The effect of MRP addition on crystallinity is considerable, but crystallinity was found to decrease significantly after the incorporation of compatibilizers into the composites. *X*_c_ for reactive compatibilizers is noticeably lower than that observed when using the nonreactive compatibilizers, which is ascribed to the LCB formation.^75,76^ Thermal properties obtained from differential scanning calorimetry and thermogravimetric characterization of *r*PET/MRP composites and neat *r*PET are summarized in Table 2 (see Table 3 for composite compositions).

**Figure 5:**
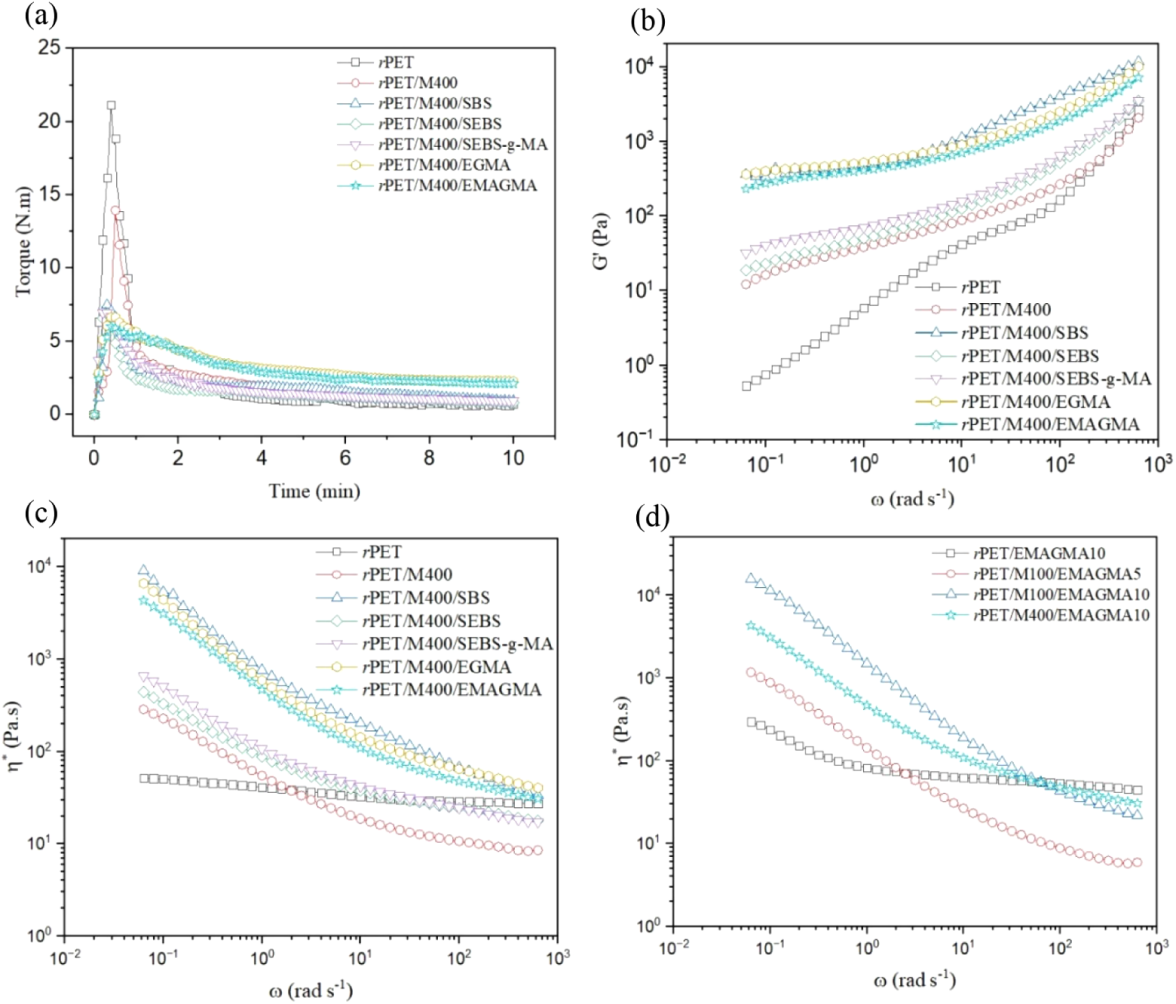
(a) Variation of torque as a function of time over melt processing, (b) storage modulus (*G’*) as a function of frequency (ω), and (c–d) complex viscosity (η*) as a function of frequency (ω) for *r*PET/MRP composites at 265 °C (see Table 3 for sample composition).

One can acquire an estimation of viscosity variations over the melt mixing process from the torque-time diagram. Typically, straight lines with negative slopes appear after 0.5–1 min of mixing (Figure 5a). After melting the thermoplastic components (i.e., *r*PET and compatibilizers) and dispersing the MRP particles in the matrix, the viscosity decreases, which is evident from the negative slope. It is worth noting that the formation of branched structures in the case of reactive compatibilizers results in relatively smaller slopes of torque reduction and the reaching of a plateau, which is not observed in the presence of nonreactive compatibilizers.^74^ In other words, the increase in the torque plateau region when reactive compatibilizers are used is due to a broadening of the molecular weight (MW) and MW distribution. In this view, the higher torque plateau for the EMAGMA-compatibilized blend compared to SEBS-*g*-MA can be attributed to the greater activity of epoxy groups compared to anhydrides in forming LCBs.^77^ Such an observation is well consistent with superior mechanical properties and relatively higher *T*_g_ for composites compatibilized with EMAGMA (Tables 1 and2).

Several factors should be considered concurrently to optimize the processability of *r*PET/MRP composites for the desired applications. We investigated the effect of various compatibilizers, MRP particle size, and compatibilizer content on the viscoelasticity and viscosity of the composite melt (Figure 5b–d). Oscillatory shear measurements were performed in the linear viscoelastic region. As seen in Figure 5b, the incorporation of 20 *wt*% MRP into *r*PET has significantly altered its terminal low-frequency behavior. The nonterminal behavior of composites is associated with the restricted long-range motion of *r*PET chains after MRP network formation in the polymer matrix. This trend has been reported in previous studies on filled polymer systems, where strong filler–polymer interactions and improved dispersion lead to network formation and suppressed terminal behavior.^78,79^ The nonterminal behavior was found to become more pronounced in the presence of compatibilizers, which implies their role in the MRP dispersion to form a network. Similar to previous findings on compatibilized polymer blends, branching by increasing polymer chain entanglements leads to increased elasticity of the polymer melt.^80^

The substantial increase (2–3 orders of magnitude) in storage modulus (*G’*) suggests that our compatibilized composites exhibit significantly higher elasticity and melt strength compared to neat *r*PET and uncompatibilized *r*PET/MRP.^81^ It is noteworthy that terminal behavior disappeared in the presence of SBS, EGMA, and EMAGMA, and a solid-like behavior in the low-frequency region is observed. Prior research on long-chain branched (LCB) PET has demonstrated that increased chain entanglements contribute to improved melt elasticity and processability, which aligns with our results.^82^

Figure 5c illustrates that the complex viscosity (η*) of the *r*PET homopolymer remains nearly independent of frequency, which is characteristic of Newtonian behavior. However, the incorporation of fillers and compatibilizers results in non-Newtonian behavior, increased elasticity, and shear-thinning properties—features commonly reported in polymer nanocomposites and compatibilized blends.^83^ It has been shown that polymers with branched chains may have viscosities 2 orders of magnitude higher than corresponding polymers with linear chains at low shear rates.^84^ At low frequencies, η* of compatibilized and uncompatibilized *r*PET/MRP composites is noticeably higher than that of *r*PET. However, at high shear rates, viscosity reduces sharply due to the disentanglement of polymer chains. A molecular structure with LCBs can explain this substantial shear-thinning behavior in modified composites.^85^ EMAGMA and EGMA-compatible *r*PET/MRP composites displayed a higher η* of approximately 1 order of magnitude compared to uncompatibilized composites, resulting in a high density of branched structures. This increase in viscosity at low shear rates has been previously observed in LCB-modified PET and is desirable for applications requiring high melt strength, such as extrusion.^86^ Furthermore, as shown in Figure 5d, increasing EMAGMA content from 5 to 10 wt % resulted in an order-of-magnitude increase in η* at low frequencies. This trend is consistent with studies on reactive compatibilization, where higher compatibilizer concentrations lead to improved interfacial interactions and branched architectures.^87^ This finding is useful for formulating *r*PET/MRP compounds with desired rheological properties for different processes.^88^

**Figure 6:**
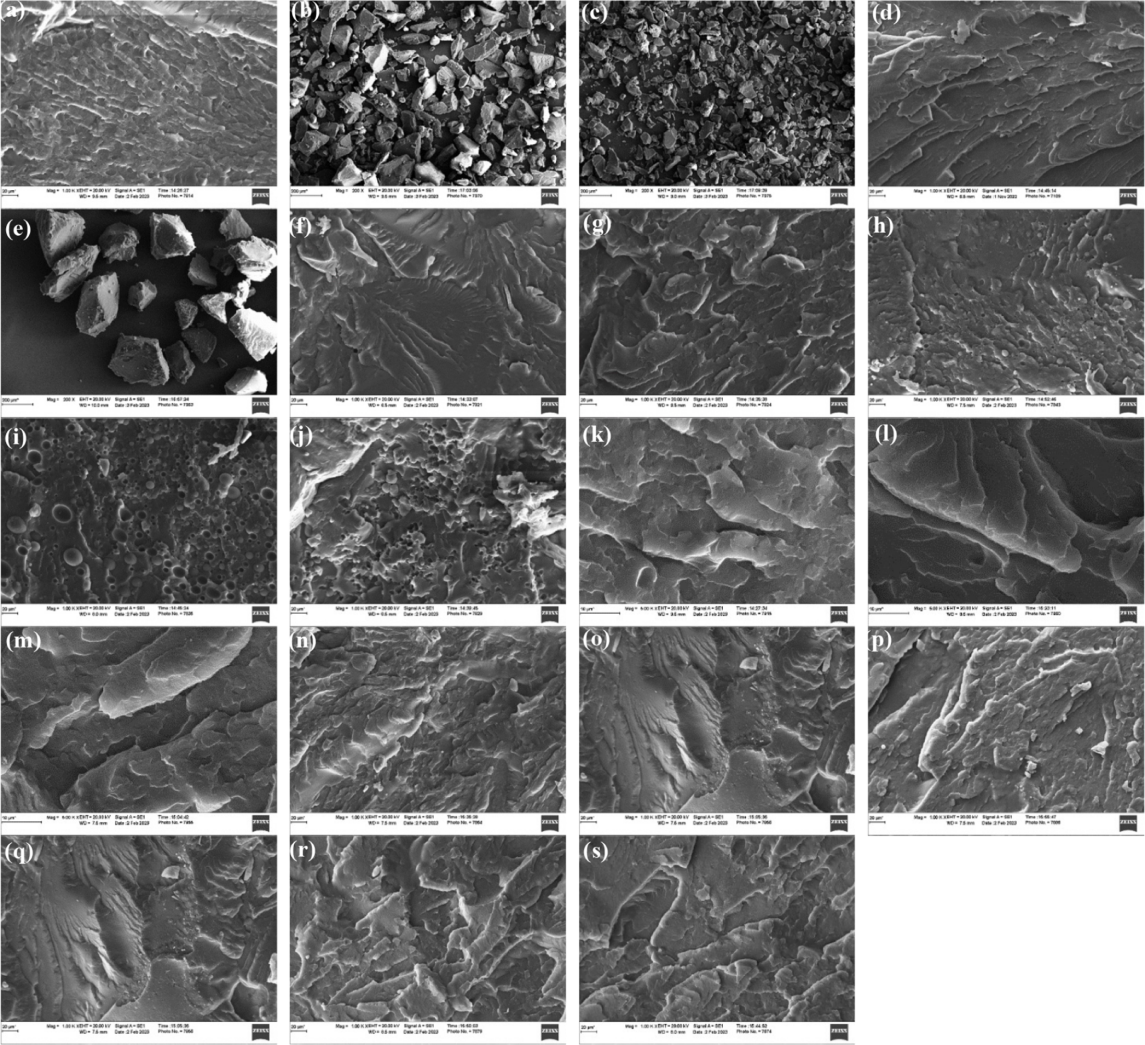
Scanning electron micrographs of (a) M400, (b) M186, and (c) M100 micronized rubber particles and cryogenically fractured surface of (d) *r*PET, (e) *r*PET/M400, (f) *r*PET/M400/EMAGMA, (g) *r*PET/M400/EGMA, (h) *r*PET/M400/SEBS-*g*-MA, (i) *r*PET/M400/SEBS, (j) *r*PET/M400/SBS, (k) *r*PET/M400/EMAGMA, (l) *r*PET/M185/EMAGMA, (m) *r*PET/M100/EMAGMA, (n) *r*PET/M100/EMAGMA5, (o) *r*PET/M100/EMAGMA10, (p) *r*PET/M100/EMAGMA10 (3), (q) *r*PET/M100/EMAGMA10 (4), (r) *r*PET/M100/EMAGMA10 (5), and (s) *r*PET/M100/EMAGMA10 (6) (see Table 3 for sample composition).

Dispersion and the interfacial adhesion of MRP particles in the *r*PET matrix were examined by scanning electron microscopy (SEM). As can be seen in Figure 6a–c, the surface of some MRP particles is porous, while others have smooth angular surfaces. Comparing the micrographs of the cryogenically fractured surface of *r*PET and *r*PET/M400 reveals that the detection of the rubber phase in the blend is difficult (Figures 6d,e). However, a more textured surface of the blend sample indicates an even distribution of MRP particles, while cracks and pores entail a weak interaction within the *r*PET matrix. Weak interfaces are more likely to cause mechanical failure under stress and make a material more susceptible to environmental and mechanical degradation. The interfacial bonding among components intensely affects fracture behavior in multicomponent composites. Thus, improved interfacial compatibility outcomes fail to occur in the continuous phase rather than at the interface. Figures 6f–h illustrates the effect of reactive compatibilizers on the morphology of composites. The relatively less textured morphology could be ascribed to the encapsulation of the rubber particles by the compatibilizer, owing to the lower interfacial tension of the MRP/compatibilizer, which is well consistent with the mechanical and rheological results discussed above. A similar observation has been reported for HDPE/SRP/elastomer composites (SRP: scrap rubber powder), where it was predicted that the lower interfacial tension of SRP/ethylene-octylene copolymer compared to HDPE/SRP can lead to SRP encapsulation by the compatibilizer.^89^ Comparing the surface morphology of *r*PET/MRP compatibilized with nonreactive SBS and SEBS copolymers implies relatively less compatibility of rubber particles with the *r*PET matrix in the presence of SEBS (Figure 6i,j). This observation agrees with the rheological results presented in Figure 5. The relatively higher compatibility of the *r*PET/MRP blend comprising SBS could be ascribed to an increase in chain mobility in the *r*PET amorphous phase due to partial plasticization by flexible polybutadiene blocks in the SBS copolymer.^90^ In another study, it has been shown that ethylene vinyl acetate, as a compatibilizer, can encapsulate cross-linked rubber particles and create an improved interface with the recycled HDPE matrix to produce thermoplastic elastomers.^42^ Typically, particle size and specific surface area play a key role in the compatibility of phases in a multicomponent blend.

As seen in Figure 6k,l, the fracture surface roughness of EMAGMA-compatibilized *r*PET/MRP composites indicates that decreasing the average particle size of MRP particles requires a higher detachment energy, which is consistent with the mechanical properties (Figure 2b). It is noteworthy that the smaller the MRP particles, the higher the interfacial adhesion as a result of better encapsulation of the MRP particles by the compatibilizer phase. The effect of the EMAGMA content on the morphology of *r*PET/MRP composites is displayed in Figure 6n,o.

It can be seen that increasing compatibilizer homogeneity in the blend is due to higher encapsulation of rubber particles by the compatibilizer and improved affinity with the *r*PET matrix. Figure 6p–s shows that by increasing the *r*PET:MRP ratio while keeping the content of EMAGMA constant, the interfacial adhesion and consequently the homogeneity of phases are increased.

**Figure 7:**
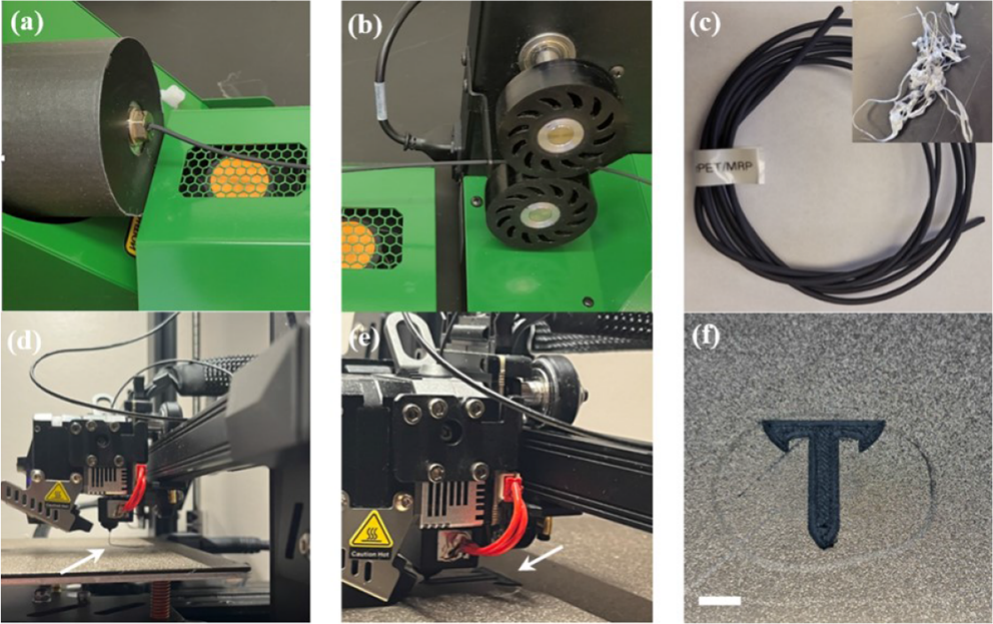
Filament extrusion and fused filament fabrication (FFF): (a) Extrusion of *r*PET/M100/EMAGMA10 composite into filament and cooling by airpath, (b) spooling the extrudate using a filament winder system, (c) produced filament (the inset picture depicts unfilled *r*PET extrudate), (d) extrusion of the prototype filament through the nozzle, (e) FFF 3D printing of the Troy University logo, and (f) additively manufactured Troy Trojans logo (the photographs in this figure were taken by Deacon S Godfrey).

Additive manufacturing (AM) techniques hold promise for sustainability owing to their capability for the rapid and on-demand fabrication of intricate objects at low cost. Fused deposition modeling (FDM), also known as fused filament fabrication (FFF), is one of the most widespread AM technologies now available with low-budget 3D printers on the market. FFF works based on the additive layer-by-layer extrusion of a thermoplastic filament through a nozzle at an elevated temperature. Generally, the high melting temperature and melt flow of postconsumer PET make its extrusion into filaments, as a feedstock for FFF, complicated. Figure 7a–c demonstrates the extrusion of EMAGMA-compatibilized *r*PET/MRP into filaments, enabled through the significantly elevated melt viscosity of the blend compared to that of the unfilled *r*PET (see Figure 5). As can be seen in Figure 7d–f, the produced *r*PET/MRP blend filaments demonstrated promising potential as sustainable feedstocks for FFF using low-budget desktop 3D printers.

## Conclusions

In this study, the effects of a series of compatibilizers (SBS, SEBS, SEBS-*g*-MA, EGMA, and EMAGMA; see Scheme 1 for the chemical structure of compatibilizers) on the thermal, mechanical, rheological, and morphological features of polyethylene terephthalate (*r*PET) and micronized rubber particle (MRP) composites were investigated. While all compatibilizers improved in mechanical properties in terms of elongation at break and impact strength compared to the uncompatibilized *r*PET/MRP blend, the best results were obtained for poly(ethylene-*co*-methyl acrylate-*co*-glycidyl methacrylate) (EMAGMA) as a reactive compatibilizing agent. Morphological characterization of composites through scanning electron microscopy revealed encapsulation of the rubber particles by the compatibilizer phase due to the lower interfacial tension with the MRP particles. A nonterminal rheological behavior was observed for *r*PET comprising 20 *wt*%, which was found to become more pronounced in the presence of compatibilizers. EMAGMA-compatibilized *r*PET/MRP composites displayed a higher complex viscosity of about 1 order of magnitude compared to the uncompatibilized blend, which is ascribed to long-chain branching. Given the superior mechanical properties along with increased melt elasticity, the extrusion of *r*PET/MRP/EMAGMA composites into filaments was enabled, demonstrating promising performance as a sustainable feedstock for fused filament fabrication 3D printing. Low-cost desktop 3D printers can produce cost-effective rPET/MRP objects containing up to 20 *wt%* waste rubber.

## Experimental Section

### Materials and Compounding

Recycled poly(ethylene terephthalate) (*r*PET, grade 3000) was supplied by Ex-Tech Plastics. This rPET grade possesses relatively high impact strength (44.7 ± 10.5 kJ m^–2^) compared to typical PET grades (14–34 kJ m^–2^).^91^ Micronized rubber powders with particle sizes of 100, 185, and 400 μm were obtained from Lehigh Technologies. SBS (C3000), SEBS (C2000), and SEBS-*g*-MA (C1000, 1.4–2 *wt*% maleic anhydride) were acquired from Kraton. EGMA and EMAGMA were purchased from Sigma–Aldrich. Irganox 1010 antioxidant was supplied by BASF.

The chemical structures of the compatibilizers used are illustrated in Scheme 1. Before processing, *r*PET flakes were dried in a vacuum oven at 100 °C for 24 h to avoid hydrolysis during melt processing. All other materials were used as received without further purification.

The compounding process was performed using an internal mixer (IntelliTorque Plasticorder, Brabender CWB) with a chamber volume of 30 cm^3^. Based on the compositions summarized in Table 3, predried *r*PET flakes, MRP powder, compatibilizer granules, and 1 *wt %* Irganox 1010 (based on the total polymer) were manually premixed to ensure initial homogenization. The mixture was then loaded into the chamber and compounded at 265 °C with a screw rotation speed of 90 rpm for 10 min. Scheme 2 shows the surface modification of MRP through the interaction between the compatibilizer and *r*PET. The compatibilizer, which contains reactive functional groups, enhances interfacial adhesion by bridging the two immiscible phases and improving stress transfer across the interface.

Subsequently, the compounds were compression molded into 0.5 mm thick sheets using a Carver benchtop hot press at 265 °C and 5 MPa, followed by water bath cooling. Standard tensile and impact specimens were cut from the molded sheets according to the dimensions specified in ASTM D638 and ASTM D1822-S, respectively. The loading content and particle size of MRP were optimized according to preliminary mechanical testing results.

### Characterization Methods

#### Fourier-Transform Infrared Analysis (FTIR)

FTIR spectra of neat *r*PET, compatibilizers, and *r*PET/MRP composites were recorded by using a Spectrum Two FTIR spectrometer (PerkinElmer) in transmission mode. Spectra were collected over the wavenumber range 4000–500 cm^–1^ at a resolution of 4 cm^–1^, averaging 32 scans per sample.

#### Mechanical Characterization

Tensile tests were performed at room temperature using an Instron 5943 universal testing machine with a 10 kN load cell, following ASTM D638. A gauge length of 15 mm and an extension speed of 10 mm.min^–1^ were used. Each test specimen had an average width of 4 mm and a thickness of 0.5 mm. The average values of elastic modulus, tensile strength, and elongation at break were reported for at least five samples per compound.

Impact strength tests were carried out on unnotched specimens (average width: 3.2 mm and thickness: 0.5 mm) using a Zwick/Roell HIT25P pendulum impact tester (2.7 J) according to ASTM D1822-S. The obtained impact energy determines the energy needed to fracture a specimen by shock in tension. At least five specimens per compound were tested, and results were presented as average values.

#### Thermogravimetric Analysis (TGA)

Thermal stability was evaluated using a TGA550 analyzer (TA Instruments). Approximately 10 mg of each sample was analyzed under a nitrogen atmosphere (flow rate: 40 mL·min^–1^) over the temperature range of 30–800 °C at a heating rate of 10 °C min^–1^.

#### Differential Scanning Calorimetry (DSC)

DSC analysis was performed using a DSC 250 instrument (TA Instruments). The DSC equipment was continuously purged with nitrogen at a flow rate of 40 mL·min^–1^. To measure the crystallization and melting temperatures, as well as the degree of crystallinity, 5–10 mg of sample was initially heated from ambient temperature to 280 °C at a rate of 10 °C min^–1^ and held isothermally at 280 °C for 2 min to remove thermomechanical histories. Subsequently, samples were cooled to −40 °C at a rate of 10 min^–1^, followed by a second heating run at the same rate to reach 280 °C. The crystallization temperature (*T*_c_), melting temperature (*T*_m_), glass transition temperature (*T*_g_), enthalpy of melting (*ΔH*_m_), and enthalpy of crystallization (*ΔH*_c_) were determined.

#### Rheological Characterization

The rheological behavior of *r*PET/MRP composites was analyzed using a DHR HR-30 rheometer (TA Instruments) equipped with 25 mm parallel plates at 265 °C. Samples were prepared in disc form (diameter: 25 mm, thickness: 1 mm) by compression molding. A strain sweep test was initially performed to establish the linear viscoelastic region. Frequency sweep tests were then conducted in the range of 0.1–100 rad·s^–1^ at a constant strain of 1%.

#### Scanning Electron Microscopy (SEM)

The morphology of *r*PET/MRP composites was examined by using a Zeiss EVO 50 Variable Pressure SEM (Carl Zeiss SMT, Inc.). Specimens were fractured in liquid nitrogen to obtain brittle fracture surfaces, mounted onto aluminum stubs with carbon tape, and coated with a thin gold layer using an EMS 550 Auto Sputter Coater (Electron Microscopy Sciences). SEM images were captured at an accelerating voltage of 20 kV under high vacuum.

#### Filament Production and 3D Printing

The optimized *r*PET/M100/EMAGMA10 composite was extruded into filaments by using a Filabot EX2 single-screw extruder at 270 °C. The extrusion speed was maintained at 15 rpm, and the extrudate was air cooled by using an integrated airpath system before being wound onto spools. The filament diameter was controlled at 1.75 ± 0.05 mm.

To evaluate the printability of the produced filaments, fused filament fabrication (FFF) was carried out by using a low-budget 3D printer (Creality Ender-3). Printing parameters were as follows: nozzle temperature: 270 °C; bed temperature: 90–100 °C; print speed: 60–100 mm·s^–1^; and layer height: 0.2 mm.

## Supporting Information Available

FTIR spectra of compatibilized *r*PET/MRP composites, neat *r*PET, uncompatibilized composite, and neat compatibilizers (SBS, SEBS, SEBS-*g*-MA, and EGMA) (PDF)
