# Supplementary material for: Development of Toughened Recycled Polyethylene Terephthalate and Micronized Rubber Composites for 3D Printing Applications: Compatibilization Strategies and Performance Assessment
Source: ACS Omega. 2025 May 1;10(18):18404–18. doi: 10.1021/acsomega.4c10726 (PMC12079225; doi:10.1021/acsomega.4c10726)
Supplement: Supplementary file 1 — ao4c10726_si_001.pdf [file ao4c10726_si_001.pdf]

Development of Toughened Recycled Polyethylene Terephthalate and Micronized Rubber Composites for 3D Printing Applications: Compatibilization Strategies and Performance Assessment

Aboulfazl Barati*, Deacon S. Godfrey, Erfan Dashtimoghadam

Center for Materials and Manufacturing Sciences, Departments of Chemistry and Physics, Troy University, Troy, AL 36082, USA

* Corresponding author. Email: abarati@troy.edu

Supporting Information

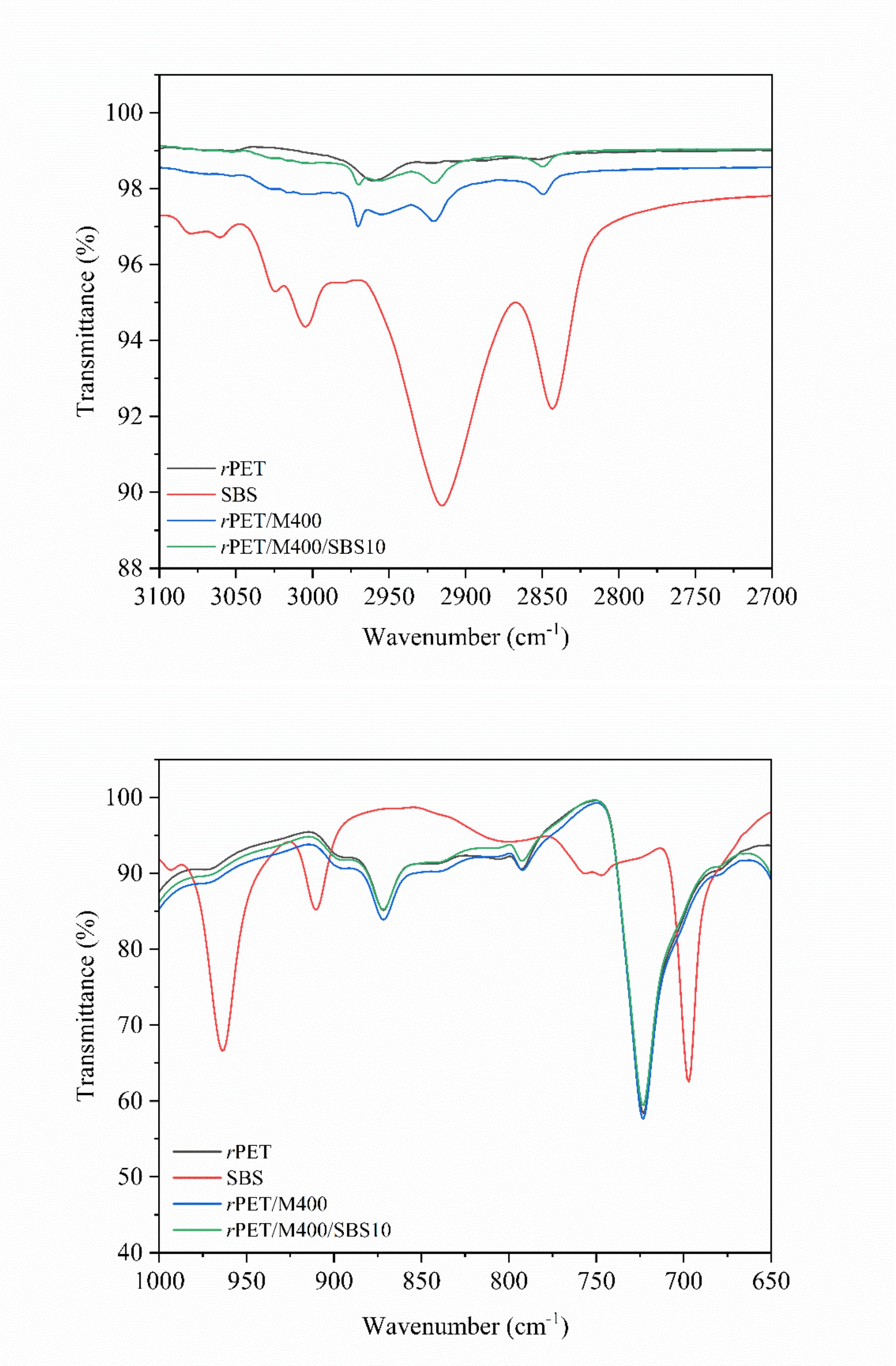

Figure S1. Fourier-transform infrared spectra of compatibilized *r*PET/MRP composites in comparison with neat *r*PET, uncompatibilized blend, and SBS as a compatibilizer, over different wavenumber range.

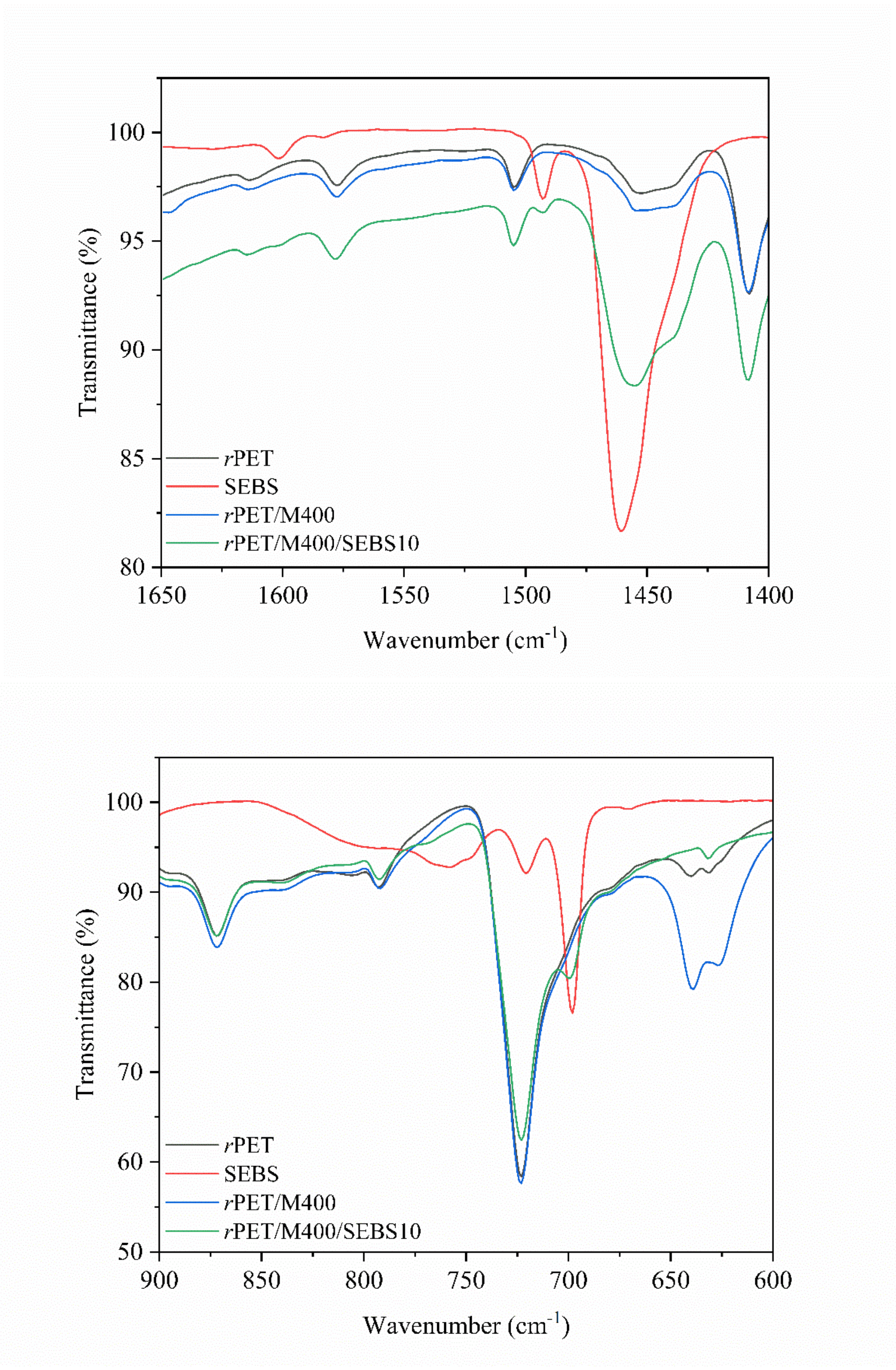

Figure S2. Fourier-transform infrared spectra of compatibilized *r*PET/MRP composites in comparison with neat *r*PET, uncompatibilized blend, and SEBS as a compatibilizer, over different wavenumber range.

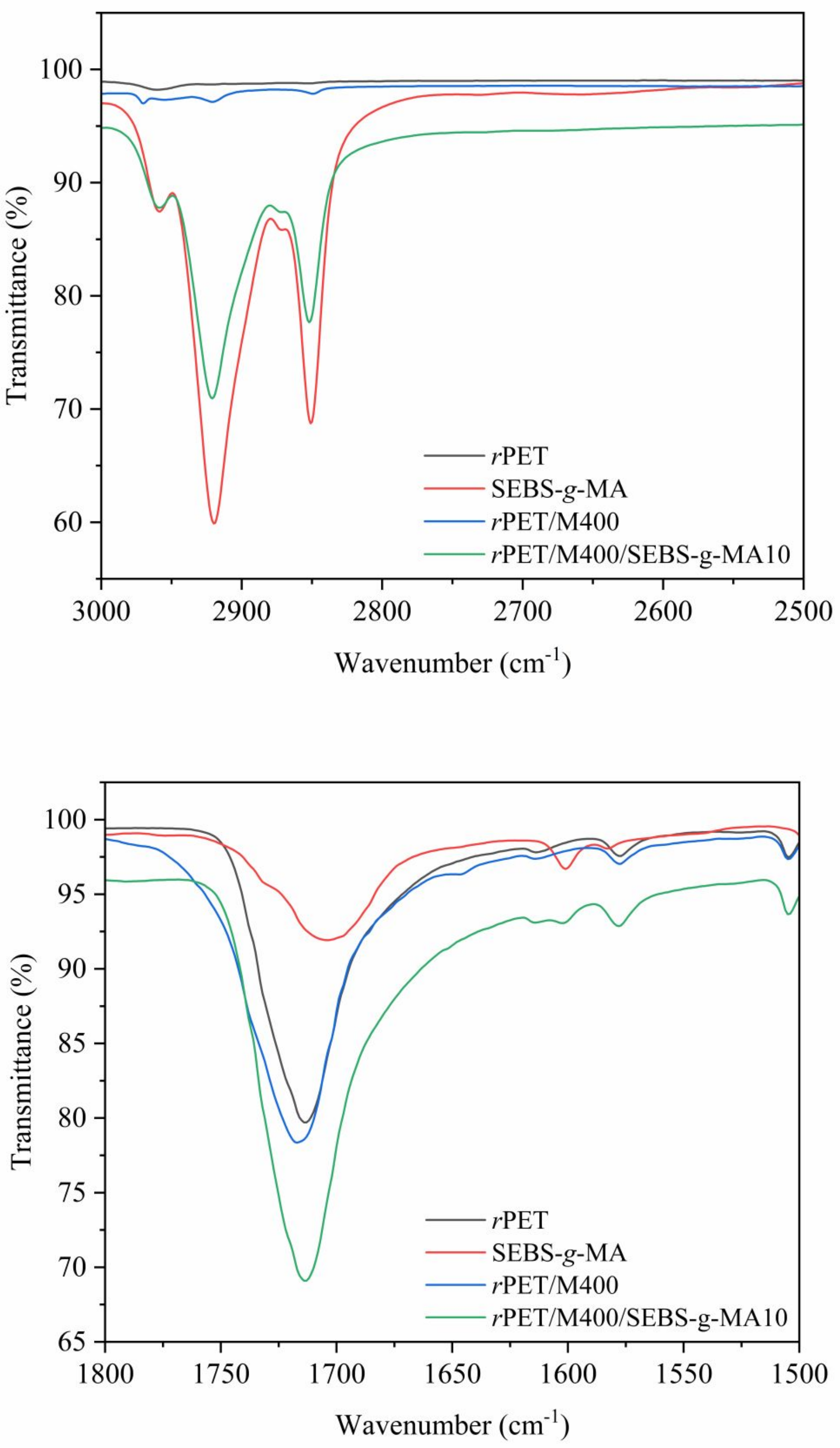

Figure S3. Fourier-transform infrared spectra of compatibilized *r*PET/MRP composites in comparison with neat *r*PET, uncompatibilized blend, and SEBS-*g*-MA as a compatibilizer, over different wavenumber range.

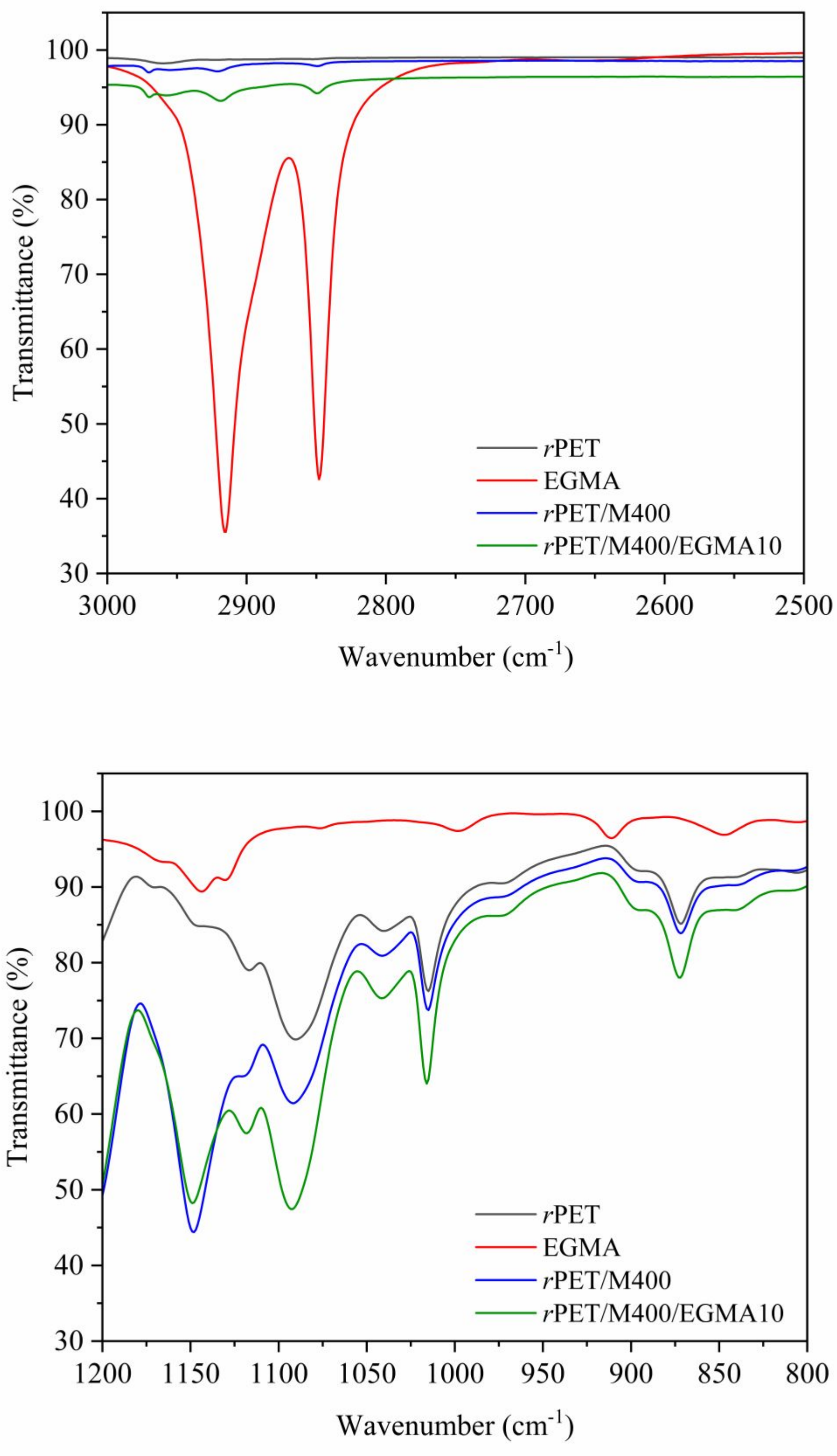

Figure S4. Fourier-transform infrared spectra of compatibilized *r*PET/MRP composites in comparison with neat *r*PET, uncompatibilized blend, and EGMA as a compatibilizer, over different wavenumber range.
